# The Highs and Lows of ADAMTS13 Activity

**DOI:** 10.3390/jcm13175152

**Published:** 2024-08-30

**Authors:** Rebecca J. Shaw, Simon T. Abrams, Samuel Badu, Cheng-Hock Toh, Tina Dutt

**Affiliations:** 1 Liverpool University Hospitals NHS Foundation Trust, Liverpool L7 8YE, UK; r.shaw3@nhs.net (R.J.S.); samuel.badu@liverpoolftt.nhs.uk (S.B.); toh@liverpool.ac.uk (C.-H.T.); tina.dutt@liverpoolft.nhs.uk (T.D.); 2 Clinical Infection, Microbiology and Immunology, Institute of Infection, Veterinary & Ecological Sciences, University of Liverpool, Ronald Ross Building, 8 West Derby Street, Liverpool L69 7BE, UK

**Keywords:** ADAMTS13, TTP, vWF, recombinant

## Abstract

Severe deficiency of ADAMTS13 (<10 iu/dL) is diagnostic of thrombotic thrombocytopenic purpura (TTP) and leads to accumulation of ultra-large vWF multimers, platelet aggregation, and widespread microthrombi, which can be life-threatening. However, the clinical implications of a low ADAMTS13 activity level are not only important in an acute episode of TTP. In this article, we discuss the effects of low ADAMTS13 activity in congenital and immune-mediated TTP patients not only at presentation but once in a clinical remission. Evidence is emerging of the clinical effects of low ADAMTS13 activity in other disease areas outside of TTP, and here, we explore the wider impact of low ADAMTS13 activity on the vascular endothelium and the potential for recombinant ADAMTS13 therapy in other thrombotic disease states.

## 1. Introduction

The year 2024 marks the passing of a century from the time when Eli Moschowitz described the initial clinical phenotype of thrombotic thrombocytopenic purpura (TTP) in a 16-year-old girl with fever, neurological symptoms, microangiopathic haemolytic anaemia, and thrombocytopenia [1]. The post-mortem histology for this index case confirmed widespread microvascular thrombotic occlusion, compromising the function of multiple vital organs and leading to death. After a series of similar cases in 1947, Singer named the distinct clinical entity “TTP” [2], however, apart from clinical manifestations and significant mortality, little more was known about the underlying pathophysiology of the condition at the time.

Despite early observations linked to the presumed existence of “A disintegrin and metalloproteinase with thrombospondin type 1 motif, member 13” (ADAMTS13), the identity of the enzyme, key to the diagnosis of TTP, remained elusive for many years. In the 1980s, Moake et al. observed the occurrence of platelet attractive ultra-large vWF (ULvWF) multimers in the plasma of relapsing TTP patients, prompting the hypothesis that patients with TTP likely lacked a depolymerase-like enzyme, that if present, would restrict the length of circulating ULvWF [3]. By 1996, Furlan et al. had purified and characterised ADAMTS13 as the metalloprotease responsible for this physiological cleavage of vWF, without which the life-threatening clinical disease TTP would ensue with inevitable morbidity and mortality unless promptly treated [4]. Effective treatment with plasma infusion and later plasma exchange had already been recognised in TTP, and identification of this critical enzyme within plasma was a key discovery representing the missing link [5].

## 2. The ADAMTS13–VWF Axis

The relationship between vWF and ADAMTS13 is sometimes referred to as the ADAMTS13-vWF axis or the ADAMTS13/vWF ratio, where the relative levels of both in the normal state can be expressed as a percentage of normal, approximately 100% or close to 1.0.

ADAMTS13 is synthesized in hepatic stellate cells and composed of an N-terminal reprolysin-type metalloprotease domain (M), followed by a disintegrin domain (D), a thrombospondin-1–like domain (T), a cysteine-rich domain (C) that contains an arginine-glycine-aspartate sequence, a spacer domain (S), seven additional thrombospondin-1-like domains (T2-8), and two nonidentical CUB-type domains (CUB1-2) at the C-terminal end of the molecule. Circulating in a “closed” formation prevents ADAMTS13 from proteolyzing unselected substrates and reduces susceptibility to inhibitors.

Under conditions of shear stress, vWF unravels from a resting globular state to expose A1 and A2 domains, hosting platelet and ADAMTS13 binding sites respectively. Exposure of the ADAMTS13 binding sites allows for a sequence of domain binding that results in the physiological actions of ADAMTS13. This commences with the induction of an “open” ADAMTS13 conformation by binding CUB1-2 with vWF D4-CK domains, triggering a sequence of binding that results in proteolysis [6]. It is this function that controls platelet-thrombogenic potential under normal conditions and explains the propensity towards microvascular thrombosis if left unchecked.

## 3. Low ADAMTS13 and Acute TTP

ADAMTS13 is today most well-known for its association with the acute diagnosis of TTP; ADAMTS13 activity <10% is pathognomonic of TTP. Reduced ADAMTS13 activity in TTP is caused by genetic mutations or inhibitory autoantibodies, leading to congenital (cTTP) or immune-mediated (iTTP), respectively [7,8]. Around 95% of patients diagnosed with TTP have immune-mediated disease [9], whilst <5% of cases are congenital [10].

Severe ADAMTS13 deficiency and the presence of inhibitory antibodies to ADAMTS13 is essential for the diagnosis of iTTP, and to differentiate it from other thrombotic microangiopathies (TMAs) [11,12]. Regular monitoring of ADAMTS13 levels during acute treatment can help evaluate clinical response to therapy and guide treatment decisions. Current treatment for acute iTTP includes ADAMTS13 replacement with therapeutic plasma exchange, immune suppression with rituximab (monoclonal anti-CD20), high-dose corticosteroids, plus inhibition of vWF-mediated platelet adhesion with the nanobody caplacizumab [13,14,15].

The measurement of ADAMTS13 activity in conjunction with genetic analysis is critical in the diagnosis and appropriate management of cTTP [16,17]. In cTTP, various mutations in the ADAMTS13 gene result in either deficient or dysfunctional ADAMTS13, and subsequent impaired cleavage of vWF multimers [16]. cTTP can present at any age; although cTTP patients have a persistently low ADAMTS13 from birth, they may remain asymptomatic until a further triggering event occurs such as pregnancy [18].

Treatment of cTTP has, until recently, focused on ADAMTS13 replacement using plasma infusion (solvent–detergent fresh frozen plasma) or factor VIII concentrates containing ADAMTS13 (BPL 8Y) [10,16,19]. These therapies are effective in the prophylactic and symptomatic management of cTTP, with treatment usually being administered every 1–3 weeks [19]. Congenital TTP patients receiving treatment with regular prophylactic donor plasma infusions to replace ADAMTS13 report symptomatic relief with commencement of prophylaxis and also have a significantly reduced incidence of stroke (2% vs. 17%) compared to those not receiving prophylaxis [17].

## 4. ADAMTS13 and TTP in Remission

ADAMTS13 activity is known to be a prognostic marker not only in the acute presentation of TTP but also in remission, with persistent undetectable levels in recovering patients associated with an increased risk of disease exacerbation or recurrence [20]. This cohort of TTP patients warrant closer monitoring for the earliest detection of an acute clinical relapse.

The ADAMTS13 activity level in remission TTP is a subject of growing interest. In particular, the population of patients achieving a clinical remission whose ADAMTS13 activity resides in the intermediate zone between low normal and severely deficient levels. George et al. described the heterogeneity of ADAMTS13 activity levels in patients classed as in a remission [21]. The group reported annual evaluations of ADAMTS13 levels between 1995 and 2014 (during a time when regular follow up and interventions were not standard practice) demonstrating natural variations in ADAMST13 activity. Authors divided patients into three categories related to their remission ADAMTS13 activity: (1) A group where remission ADAMTS13 activity was always normal (≥60%) and patients did not incur a relapse, (2) a group where remission ADAMTS13 activity was not always normal but >10%, and (3) a final group in which the ADAMTS13 activity remained 5–15%. For the latter two groups, a proportion of patients relapsed but there were also individuals who avoided acute relapse or spontaneously recovered without any intervention. The authors concluded that ADAMTS13 deficiency during remission was associated with relapse however individual profiles of ADAMTS13 activity were unpredictable and variable. Sustained ADAMTS13 deficiency may be seen without an acute TTP episode, but the potential consequences of chronic sub-normal ADAMTS13 activity levels on longer term health and risk are not clear.

Upreti et al. concluded that TTP patients in remission exhibiting a lower baseline ADAMTS13 level appear to have an increased risk of ischaemic stroke (13.1% TTP in remission versus 2.6% in the general population). In this group, further associations have been made stratifying ADAMTS13 activity in remission, specifically >70%, 40–70%, 10–39%, and <10%. These strata correlate with a 0% incidence of stroke in those with ADAMTS13 over 70% versus 27.6% for those with levels below 70% [22].

Whilst the literature often classifies TTP patients as being in “remission”, we are beginning to observe an increasing number of reports detecting subclinical biomarkers for patients whose ADAMTS13 falls below the threshold of 70% and, in some cases, <10% in the presence of normal full blood count indices. Interestingly, a recent longitudinal study by Prasannan et al. demonstrates the importance of ADAMTS13 conformation in determining risk of relapse, with an open conformation often preceding relapse. At their peak ADAMTS13 activity, patients with a closed ADAMTS13 conformation had an eight-fold lower relapse rate within 1 year (9% vs. 46%) and a five-fold lower relapse rate within 2 years (23% vs. 62%) compared to patients with an open ADAMTS13 conformation [23].

A series of recent publications has highlighted the possible connection between white matter changes on MRI, impaired neurocognitive and ADAMTS13 level in patients with TTP [24,25]. In a report by Hannan et al., of the iTTP patients defined as in “remission”, half had an ADAMTS13 activity level <70%, and one fifth had a level <10% [26]. Alwan et al. found lower than average ADAMTS13 activity levels in patients with silent cerebral infarction (half the patients studied) versus those without, and higher rates of cognitive impairment [24]. There is an increasing suggestion that patients in a clinical remission with lower-than-average ADAMTS13 activity levels may warrant earlier intervention. This is aimed at restoring ADAMTS13 activity to optimise vascular integrity to potentially retard the rate of sub-clinical cerebrovascular disease. This concept is challenging the way TTP is regarded as a condition characterised by either a distinct acute or remission state [25].

## 5. ADAMTS13 and Other Disease

The normal reference range for ADAMTS13 varies depending on the assay methodology, age, gender and genetic variants in different ethnic populations, and is usually described with a range between 40–140% of healthy adults. In TTP specialist centres, different thresholds are accepted when monitoring the progress of their patients with TTP. For example, in the UK and Europe, this is usually >40%, compared to some U.S. centres using > 70%.

Polymorphisms found at higher prevalence in certain populations can also influence normal range limits, for example, the P.Pro475Ser polymorphism known to be heterozygous in approximately 10% of the Japanese population is associated with a reduction in ADAMTS13 activity level [27].

A low ADAMTS13 activity appears to have important clinical implications outside of the field of TTP, particularly in those conditions which are recognised as being pro-thrombotic. This is in keeping with the known antithrombotic action of ADAMTS13, cleaving highly active ultra-large vWF multimers in the circulation [4]. Although extreme deficiency of ADAMTS13 is highly specific for a diagnosis of TTP, lesser reductions are observed in other thrombotic microangiopathies including haemolytic uraemic syndrome, sepsis and malignancy [28].

### 5.1. Cerebral Ischaemia

In a large study of almost 6000 participants aged ≥ 55 years in the Netherlands, Sonneveld et al. determined that a low ADAMTS13 activity was associated with increased risk of ischaemic stroke in the general population [29]. A low ADAMTS13 activity remained a significant risk factor for stroke, even after adjustment for traditional cardiovascular risk factors (including age, gender, smoking status, blood pressure, diabetes, and cholesterol). Those with an ADAMTS13 activity in the lowest quartile had a significantly higher risk of ischemic stroke than those in the highest quartile (absolute risk, 7.3% vs. 3.8% respectively; hazard ratio, 1.65; 95% confidence interval [CI], 1.16–2.32). Authors proposed that lower ADAMTS13 activity levels would lead to increased prevalence of large procoagulant vWF multimers, which could subsequently lead to development of thrombus especially at sites of high shear stress or endothelial damage [29].

With ischaemic stroke being a major cause of morbidity and mortality worldwide [30], and available treatments options being sub-optimal, there is huge potential for using ADAMTS13 as a novel therapeutic target in these patients.

It is of note that within this large study, individuals with an ADAMTS13 activity in the lowest quartile still had an activity level which would be considered to be in the normal range (>50 iu/dL) [31]. Therefore, it appears that ADAMTS13 activity does not have to be severely low, as in patients diagnosed with TTP, to still have pathological implications and potentially clinical consequences.

### 5.2. Myocardial Ischaemia

Myocardial ischaemia/infarction is a major cause of global morbidity and mortality; a meta-analysis of >3500 participants (with myocardial infarction and healthy controls) showed that ADAMTS13 level below the 5th centile was associated with a moderate increased risk of myocardial infarction (OR 1.89, [95% CI 1.15–3.12]) [32]. The risk was similar between different age groups but appeared more pronounced in women compared to men, although not all confounding factors were accounted for. Unlike in ischaemic stroke, moderately low ADAMTS13 levels were not associated with an increased risk of myocardial infarction (lowest quartile vs. highest quartile, OR 1.28 [95% CI 0.68–2.45]), and there was no trend observed in the intermediate quartiles, with the authors suggesting a clinically relevant “threshold” for ADAMTS13 activity and increased myocardial ischaemia risk rather than a dose-dependent relationship [32].

The pathophysiology of low ADAMTS13 activity being associated with myocardial infarction is not fully understood. Suggested mechanisms include an effect of ADAMTS13 on the initiation and progression of atherosclerotic plaques [33], its effect on acute arterial thrombus formation, and/or the amplification of a developing thrombus [34,35].

### 5.3. Renal Disease

The effect of ADAMTS13 deficiency on cardiovascular outcome has also been reported in patients with end-stage renal failure on haemodialysis (HD). One study demonstrates significantly reduced ADAMTS13 activity in HD patients compared to normal healthy controls (mean ADAMTS13 41.0 ± 22.8% vs. 102.3 ± 17.7%, respectively), which was an independent risk factor for the development of new cardiovascular events in this group of patients. The cause for the low ADAMTS13 activity in this cohort is not known, although it has been hypothesised that there is a potential role for synthesis of ADAMTS13 from the kidneys, which is therefore reduced in chronic kidney disease [36], and it is recognised that renal failure patients often have other comorbidities which may contribute. Ocak et al. showed an increased mortality in 956 dialysis patients with higher levels of vWF and reduced ADAMTS13 activity; specifically, those dialysis patients in the highest quartile of vWF had a 1.4-fold (95% CI 1.1–1.8) increased mortality risk compared with those in the lowest quartile for vWF, even after adjustment for other risk factors including age, sex, body mass index, cardiovascular disease, smoking, type of dialysis, primary kidney disease, use of antithrombotic medication, blood pressure, albumin levels, CRP levels and baseline eGFR [37]. The lowest quartile of ADAMTS13 activity (mean level 16.7%) was associated with a 1.3-fold (95% CI 1.0–1.7) increased mortality risk after adjustment, compared with the highest quartile of ADAMTS13 activity (mean level 68.1%). Different causes for mortality in these patients were explored, and overall high vWF levels and low ADAMTS13 activity were associated with a 1.8-fold (95% CI 1.0–3.2) increased cardiovascular mortality risk and a 2.1-fold (95% CI 1.2–3.7) increased non-cardiovascular mortality risk [37], with the increased mortality speculated to be likely due to an increased prothrombotic tendency.

### 5.4. COVID-19 Infection

The COVID-19 pandemic has had a hugely significant global impact since 2020 [38]. While little was known early on about this novel virus, reports soon emerged of the prothrombotic nature of SARS-CoV-2 infection, with patients at increased risk of developing thromboses acutely and even post-discharge from hospital [39,40,41,42].

COVID-19 infection has been shown to be associated with a reduction in activity of ADAMTS13, which was particularly seen in those with severe infection [43]. As expected, a decrease in ADAMTS13 activity leads to an increase in vWF concentration and activity and alteration of the vWF:ADAMTS13 ratio in this carefully balanced axis. Mancini et al. found a moderate reduction in ADAMTS13 activity in the more severe cases of COVID-19 infection; around one-third of patients in critical care presented with ADAMTS13 activity levels of ˂50 iu/dL. They also observed a 3–7-fold increase in the vWF antigen:ADAMTS13 activity ratio associated with the severity of COVID-19 infection [44]. This study found a slight decrease in high-molecular-weight vWF multimers and a relative increase of intermediate/low-molecular-weight vWF multimers, which was more pronounced in the most severe cases of COVID-19 infection; authors proposed that this may be explained by an early increase of vWF proteolysis by ADAMTS13, as it attempts to overcome the excess release of vWF in response to local hyperinflammation and endothelial activation in COVID-19. The theory is that ADAMTS13 will be “consumed” during this process, tipping the balance in the ADAMTS13-vWF axis and reaching reduced levels in the most critically ill patients. High molecular weight multimers may also be consumed in vWF-platelet aggregates and microthrombi, similar to the pathophysiology seen in the acute presentation of TTP.

As well as an association with severity of infection, ADAMTS13 activity has been shown in several studies to be associated with mortality in COVID-19. A study of 88 PCR-proven COVID-19 patients demonstrated that COVID-19 non-survivors had significantly lower levels of ADAMTS-13 activity (32.2 iu/dL vs. 50.6 iu/dL, *p* = 0.035) and higher levels of vWF (395.5 iu/dL vs. 295.5 iu/dL, *p* = 0.033) when compared to patients who survived [45]. Looking at ratios, a vWF:RCo/ADAMTS13:activity ratio of >5.7 was associated with ICU admission, and a ratio >6.5 was associated with increased patient mortality [46] in COVID-19 infection.

The implications of low ADAMTS13 activity in COVID-19 have also been shown in pregnancy, with an increased vWF RiCof:ADAMTS13 activity ratio being significantly associated with a higher risk of pregnancy-related complications including pre-term delivery (OR 1.9, 95% CI 1.1–3.5). The placentae of women with COVID-19 infection show inflammatory histological features, with increased vWF expression in the endothelium, particularly in severe cases [47].

Studies suggest ADAMTS13 deficiency may also play a role in the long-term complications of COVID-19 or “Long COVID”, which is estimated to affect 30–40% of individuals after infection with SARS-CoV-2. Prasannan et al. found an abnormal vWF:ADAMTS13 ratio > 1.5 correlated with patients with long COVID and impaired exercise capacity [48] (OR 4). Additionally, a study of 50 patients at least 6 weeks post-infection with COVID-19 showed plasma ADAMTS13 levels were significantly reduced when compared to healthy controls (Lower limit of normal local reference for the study 399 ng/mL [Median 598 ng/mL vs. 630 ng/mL, *p* = 0.009]) [49]. ADAMTS13 levels were also significantly lower in convalescent patients who had required hospitalisation when compared to those who had been managed as outpatients (*p* = 0.04) [49]. Reduced ADAMTS13 levels were seen alongside elevated vWF antigen levels in the convalescent patients, leading to an increased vWF:ADAMTS13 ratio [49] and axis imbalance.

### 5.5. Sepsis

As microangiopathic changes are not uncommon in patients with sepsis, it is unsurprising to find several studies confirming the finding of low ADAMTS13 levels [50,51,52]. Low ADAMTS13 activity is a clinically relevant finding as the magnitude of the decrease in ADAMTS13 is strongly correlated with adverse outcomes in sepsis; approximately a third of patients with sepsis have ADAMTS13 activity levels that are less than 50% of normal [52,53,54]. Furthermore, sepsis patients with such low levels have an ~10% higher risk of death compared with patients who present with no/mild reductions in ADAMTS13 activity levels [55]. Notably, the level of ultra-large vWF multimers in patients with sepsis is inversely correlated with the ADAMTS13 level [51]. These findings may be from a combination of increased consumption of ADAMTS13, its reduced synthesis, proteolytic clearance by thrombin, plasmin and/or leucocyte elastase, or the presence of proinflammatory cytokines that inhibit ADAMTS13, e.g., IL6.

### 5.6. Heparin-Induced Thrombocytopenia (HIT)

ADAMTS13 deficiency has also been associated with other thrombotic disease processes. HIT is a potentially life-threatening thrombotic complication that can occur after administration of heparin, or rarely, as an autoimmune phenomenon. It is driven by the formation of antibodies against platelet factor 4 (PF4)- heparin complexes [56,57]. Chan et al. analysed 261 patients with suspected HIT, of which one-third were confirmed positive for HIT antibodies by enzyme immunoassay [58]. They found a significant difference in ADAMTS13 activity between healthy volunteers and those with confirmed HIT, there was also a significantly lower ADAMTS13:vWF antigen ratio in the patients with HIT. In terms of clinical relevance, a multivariate analysis demonstrated that an ADAMTS13 activity of <50 iu/dL was associated with an increased risk of 90-day mortality in all patients with suspected HIT, regardless of the HIT antibody results, however the predictive value was better in those with confirmed positive antibody testing [58].

### 5.7. Sickle Cell Disease

Sickle cell disease is a haemoglobinopathy resulting from a single amino-acid mutation of the beta-globin chain of haemoglobin and can be complicated by vaso-occlusive crises, acute chest syndrome and multi-organ failure. Thrombotic complications can also include venous thrombosis and stroke. Although Demagny et al. did not find any significant reduction in ADAMTS13 antigen in patients with sickle cell compared to healthy controls, nor any difference in ADAMTS13 antigen between sickle cell patients in chronic steady state vs. those with veno-occlusive crises/acute chest syndrome [59], significant differences have been seen in vWF antigen, with increased levels in patients presenting with vaso-occlusive crisis [59,60,61] and elevated levels in more severe genotypes (HbSS or HBSβ0) [62], suggesting dysregulation of ADAMTS13-vWF axis. Fogarty et al. demonstrate that despite treatment with hydroxycarbamide or blood transfusion, a proportion of children with sickle cell disease (HbSS) had persistently elevated vWF antigen and activity, as well as elevated vWF propeptide consistent with acute endothelial cell activation [63]. Sickle cell disease mice models appear to show a benefit of recombinant ADAMTS13; administering recombinant ADAMTS13 to these mice reduced hypoxia-reoxygenation induced haemolysis as well as systemic/local inflammation in lungs and kidneys. Colombatti et al. demonstrate that patients with sickle cell disease and silent cerebral infarcts, have a significant decrease in ADAMTS13 antigen levels [64]. Together, this suggests that recombinant ADAMTS13 may be an effective novel therapy for sickle cell-related acute events and reducing organ damage [65].

### 5.8. Solid Organ Transplant

Reduced ADAMTS13 activity (hence reduced cleavage of vWF) has been implicated in both liver and lung transplant failures [66,67]. Furthermore, recombinant human ADAMTS13 therapy has been shown to significantly improve duration of skin allograft survival in mice models [68]. Although the mechanism by which recombinant ADAMTS13 therapy had effect was not determined, authors postulated this could be due to disruption of neutrophil extracellular traps (NETs) within the graft or reduction in NETs formation due to lack of ULvWF. The role of NETs within the ADAMTS13-vWF axis and relevance within other prothrombotic diseases is an evolving field.

## 6. Recombinant ADAMTS13 Therapy

Hypothetically, restoring ADAMTS13 levels and/or reducing ULvWF multimer levels may be effective treatment in a variety of disease states.

Recombinant human ADAMTS13 (rhADAMTS13) has been evaluated in animal models of thrombotic microangiopathy. Mouse, rat and baboon models were originally utilised to determine the pathophysiology of TTP [69,70]. As well as providing a deeper understanding of TTP, these animal models have proven invaluable in the development and evaluation of recent novel therapies, including recombinant human ADAMTS13 [71]. ADAMTS13-deficient (ADAMTS13−/−) mice have been developed by several research groups by gene targeting using a mixed-strain C57BL/6J-129X1/SvJ genetic background. ADAMTS13 knock-out mice do not spontaneously develop clinical evidence of TTP and have a comparable life expectancy compared to wild-type mice [72,73].

Recombinant human ADAMTS-13 has been tested in mice models of TTP. ADAMTS13 −/− mice were challenged with a high dose of recombinant human vWF (2000 units/kg). Animals rapidly developed severe thrombocytopenia and clinical symptoms of TTP. Prophylactic infusion (200 U/kg) or therapeutic infusion (320 U/kg; up to 180 min post vWF challenge) reduced disease severity [71]. Specifically, when ADAMTS13 −/− were treated with prophylactic rhADAMTS13 prior to vWF administration, no mice showed clinical, hematologic, or pathologic signs of TTP. However, therapeutic infusion of rhADAMTS13 demonstrated differing pathologic clinical and in histopathologic improvements, with the most favourable being observed at earlier time points of administration (15 min). These data highlight the benefit of early administration of rhADAMTS13 in mice models of TTP.

Tersteeg et al. developed a rat model by injecting polyclonal anti-ADAMTS13 antibodies, additional infusion of recombinant vWF was required to induce the clinical syndrome of TTP. In addition, the TTP syndrome was short-lasting, and rats began to recover after 24 h. Recombinant ADAMTS13 at doses between 400–1600 U/kg (injected into the rats 15 min after TTP symptoms were triggered) prevented cytopenias and the rise in LDH and reduced microthrombi in organs compared to control rats [74].

The most advanced evidence is in patients with congenital TTP (cTTP). A Phase III trial evaluating recombinant ADAMTS13 (TAK-755) replacement therapy for cTTP facilitated a five-fold increase in ADAMTS13 activity levels compared to patients receiving plasma-based therapy [75]. The incidence of thrombocytopenia was reduced by 60 percent in recipients of recombinant human ADAMTS13 (rhADAMTS13) and the drug was found to be safe, non-immunogenic and well tolerated. Recombinant ADAMTS13 was FDA approved in November 2023 for prophylaxis and on-demand treatment of adult and pediatric patients with cTTP. There is now considerable interest on its potential for improving outcomes in acquired deficiency states, as described above. In particular, trials assessing whether recombinant ADAMTS13 may have a role in the acute presentation of iTTP and affect the need for plasma exchange are currently being investigated. There is also an ongoing clinical trial investigating the benefit of recombinant ADAMTS13 therapy in sickle cell disease, which showed no safety signals or dose-limiting toxicities, such as bleeding events, in the Phase I placebo-controlled study [76].

## 7. Conclusions

The role of ADAMT13 in disease states, not least TTP, has entered a new era. We not only seek to confirm a severe deficiency of ADAMTS13 activity level in suspected acute presentations but now also regularly monitor levels life-long to predict relapse. The clinical phenotype of TTP is no longer perceived as two ends of a spectrum represented by distinct acute and remission states, but a disease where resting ADAMTS13 levels may predict vascular health in the mid-long term for patients with congenital and immune-mediated TTP. The evolving literature of the role of ADAMTS13 in other prothrombotic health conditions, affecting larger numbers of the population experiencing myocardial ischaemia, cerebrovascular disease and infections such as COVID-19, confirm the importance of this enzyme in regulating haemostasis, and its value as a target for novel therapies. Recombinant ADAMTS13 therapy is set to become an integrated component of management for cTTP and potentially iTTP in the near future, with potential benefits for both patient outcome and quality of life. With increasing scrutiny of the effects of fluctuating ADAMTS13 activity levels, the value of recombinant ADAMTS13 therapy may extend to the group of patients who experience sub-optimal ADAMTS13 level recovery. The role of ADAMTS13 outside the traditional context of rare disease is increasingly being characterised, broadening the scope for novel therapies to provide health benefits to larger disease populations at risk of microvascular thrombosis.
